# Integrating clinical knowledge to preserve clinical coherence in health systems

**DOI:** 10.3389/fsysb.2026.1866901

**Published:** 2026-06-26

**Authors:** Lawrence Merle Nelson

**Affiliations:** Mary Elizabeth Conover Foundation, Inc., McLean, VA, United States

**Keywords:** artificial intelligence in healthcare, clinical coherence, clinical knowledge integration, clinical phenomenological data, continuity of care, duty of care governance, health systems, primary ovarian insufficiency

## Abstract

Modern health systems generate, store, and analyze unprecedented biological information, yet translating it into coherent longitudinal care remains difficult. Fragmentation across encounters, specialties, organizations, and digital systems contributes to delayed diagnosis, inconsistent interpretation, and management misaligned with patients’ health-related context. This paper introduces Clinical Coherence as a framework for understanding how clinically meaningful knowledge is integrated and preserved within health systems. Clinical Coherence is defined as the capacity to maintain a consistent representation of a patient’s condition over time through integration of biological knowledge, Clinical Phenomenological Data, and interpretive clinical judgment. Clinical Phenomenological Data refers to structured representations of integrated life experiences relevant to health and wellbeing, including symptoms, function, uncertainty, family systems, spiritual or existential concerns, treatment burden, and signals that shape illness perception and management. These forms of knowledge are vulnerable to fragmentation as care becomes distributed across organizational and technological boundaries. Drawing on healthcare quality, continuity of care, patient-centered medicine, Narrative Medicine, complexity science, knowledge management, and clinical governance, Clinical Coherence is conceptualized as an emergent systems property rather than an attribute of an individual clinician, encounter, or technology. Duty of Care Governance is presented as the architecture through which knowledge is preserved through sensing, responsibility, escalation, and feedback. Primary Ovarian Insufficiency serves as a sentinel condition showing how fragmentation across domains can undermine care despite biomedical knowledge. As digital health and artificial intelligence reshape healthcare, Clinical Coherence offers a framework for evaluating whether systems preserve the conditions through which information becomes knowledge and understanding becomes care.

## Introduction: The problem of clinical knowledge integration

Biological systems generate signals that acquire clinical meaning only through integration with experiential and interpretive domains of knowledge. Modern health systems have achieved unprecedented capacity to generate, store, transmit, and analyze biological and clinical data (Adler-Milstein et al., 2017; World Health Organization, 2024). Recent developments in digital health, women’s health innovation, artificial intelligence governance, and trustworthy AI implementation underscore both the promise and the unresolved risks of increasingly data-driven care (World Health Organization, 2024; Grace et al., 2025; Lekadir et al., 2025). At the same time, persistent gaps remain in the ability of health systems to translate available information into coherent longitudinal care across encounters, clinicians, and organizational boundaries (McGlynn et al., 2003; Morris et al., 2011; National Academies of Sciences, Engineering, and Medicine, 2021). Fragmented management, delayed diagnosis, inconsistent interpretation, and discontinuity of care remain common despite increasing informational sophistication (Haggerty et al., 2003; Starfield et al., 2005; Pereira Gray et al., 2018).

These failures are particularly visible under conditions of clinical complexity, where uncertainty, heterogeneity of presentation, longitudinal change, and variability in patient-reported experience place sustained demands on knowledge integration over time (Engel, 1977; Plsek and Greenhalgh, 2001). Existing frameworks, including the biopsychosocial model, patient-centered medicine, continuity of care, Narrative Medicine, and integrated care, have each emphasized important dimensions of relational, experiential, and coordinated healthcare (Engel, 1977; Mead and Bower, 2000; Haggerty et al., 2003; Charon, 2007; Epstein and Street, 2011; Valentijn et al., 2013). However, large-scale health systems increasingly operate through distributed digital, organizational, and administrative structures in which biological data, patient experience, and interpretive clinical judgment may become separated across time, settings, and decision-making layers (Henry, 2006; Greenhalgh et al., 2017; Farnese et al., 2019; Whitelock-Wainwright et al., 2022).

The challenge is therefore not simply the accumulation of information, but the preservation of clinically meaningful integration across domains of knowledge. This paper introduces Clinical Coherence as a systems-level framework for understanding how biological knowledge, Clinical Phenomenological Data, and interpretive clinical judgment remain integrated within scalable health systems. Clinical Coherence is proposed as the capacity of a health system to maintain a consistent, actionable, and longitudinally meaningful representation of a patient’s condition across encounters, clinicians, organizations, and time.

Drawing upon systems theory and complexity science, which emphasize that meaningful system behavior emerges through relationships among interacting components rather than through isolated elements (Plsek and Greenhalgh, 2001), the framework views clinical integration as a systems-level challenge. It further builds upon continuity-of-care scholarship, which has demonstrated the importance of preserving relationships, information, and management across time (Haggerty et al., 2003; Valentijn et al., 2013). The framework is also informed by knowledge management theory, which examines how information becomes actionable understanding within organizations (Farnese et al., 2019), and by clinical governance traditions that emphasize accountability, quality improvement, and organizational learning (Scally and Donaldson, 1998; Chassin and Loeb, 2013). Together, these traditions position preservation of clinically meaningful knowledge as a structural challenge arising within distributed systems of care.

Primary Ovarian Insufficiency serves as a sentinel condition through which these challenges become particularly visible. The condition is defined by impaired ovarian function before age forty and is associated with infertility, menstrual irregularity, hypoestrogenism, and long-term health risks affecting bone, cardiovascular, and psychosocial wellbeing (Nelson, 2009; Panay et al., 2024). Women with Primary Ovarian Insufficiency frequently experience delayed diagnosis, fragmented care pathways, uncertainty, and inconsistent clinical messaging despite extensive biomedical knowledge regarding ovarian function and reproductive endocrinology (Alzubaidi et al., 2002; Welt, 2008; Butts, 2025). The condition also carries substantial psychosocial burden, including fertility-related distress, disrupted expectations, and emotional vulnerability (Groff et al., 2005; Davis et al., 2010; Driscoll et al., 2016; Xi et al., 2023). As such, Primary Ovarian Insufficiency provides a clinically relevant model for examining how fragmentation across domains of knowledge may contribute to incoherent care even in information-rich healthcare environments.

### Relationship to existing models of care

Clinical Coherence builds upon, but remains distinct from, several established traditions that have shaped contemporary approaches to healthcare integration, continuity, and patient-centered practice. The framework does not seek to replace these models. Rather, it synthesizes and extends them within the context of increasingly complex, data-intensive, and digitally mediated health systems.

The biopsychosocial model established an important conceptual foundation by arguing that biological, psychological, and social dimensions of illness are inherently interconnected and cannot be adequately understood through reductionist biomedical approaches alone (Engel, 1977). Patient-centered medicine further emphasized the importance of understanding illness within the patient’s perspective and relational context (Mead and Bower, 2000; Epstein and Street, 2011). Narrative Medicine subsequently highlighted the interpretive importance of patient narratives, clinical listening, and longitudinal understanding in healthcare practice (Charon, 2007). Together, these traditions expanded medicine beyond purely biological representation and reinforced the importance of experiential and contextual dimensions of care.

Related frameworks addressing continuity and integration have focused on coordination across healthcare systems. Continuity of care literature emphasizes informational continuity, relational continuity, and coordinated management over time (Haggerty et al., 2003; Pereira Gray et al., 2018). Integrated care models similarly examine how organizational, professional, and clinical coordination influence health system performance and patient outcomes (Valentijn et al., 2013). These approaches have contributed substantially to understanding fragmentation within modern healthcare delivery and have informed efforts to improve communication, transitions, and coordination across systems.

Clinical Coherence also intersects with established traditions in healthcare quality, implementation science, and learning health systems. Donabedian’s structure-process-outcome framework emphasized that healthcare quality emerges through relationships among organizational structures, clinical processes, and patient outcomes (Donabedian, 1988). Learning Health System models similarly focus on the continuous generation and application of knowledge to improve care over time (Institute of Medicine, 2001; National Academies of Sciences, Engineering, and Medicine, 2021). More recently, Greenhalgh et al. presented a framework highlighting the challenges of sustaining innovation across complex adaptive systems characterized by interacting technological, organizational, and human factors (Greenhalgh et al., 2017). Clinical Coherence complements these approaches by focusing specifically on whether clinically meaningful knowledge remains integrated as information moves across structures, processes, technologies, and organizational boundaries.

Clinical Coherence aligns with these traditions while addressing a distinct systems-level question: whether clinically meaningful knowledge remains sufficiently integrated to support coherent understanding and action across time. Existing frameworks have substantially advanced understanding of patient-centeredness, continuity, coordination, quality, and organizational learning. Clinical Coherence does not seek to replace these approaches. Rather, it focuses on the preservation of relationships among biological knowledge, Clinical Phenomenological Data, and interpretive clinical judgment as information moves across encounters, clinicians, organizations, and technologies.

This distinction becomes increasingly important as healthcare systems adopt digital infrastructures, algorithmic decision-support tools, artificial intelligence, and highly structured data architectures (Adler-Milstein et al., 2017; Topol, 2019; Lekadir et al., 2025). These technologies may improve information processing and scalability while simultaneously increasing the risk that experiential, contextual, and interpretive dimensions of care become fragmented across systems optimized for classification, prediction, and transactional efficiency (Floridi et al., 2018; Amann et al., 2020). Clinical Coherence therefore focuses on the preservation of clinically meaningful integration rather than the accumulation or transmission of information alone.

Clinical Coherence is proposed as a knowledge-integration construct operating within existing systems of care. Its purpose is not to introduce another model of healthcare delivery, but to provide a framework for examining whether clinically relevant knowledge remains sufficiently connected to support coherent longitudinal understanding of the patient. This distinction requires a more precise definition of Clinical Coherence as a systems-level construct.

### Defining clinical coherence

The challenge of integrating diverse forms of clinical knowledge has been recognized throughout the evolution of modern medicine. Engel’s biopsychosocial model argued that effective care requires integration of biological, psychological, and social dimensions of illness rather than reliance on biological information alone (Engel, 1977). Patient-centered medicine emphasized the importance of understanding illness within the patient’s perspective and relational context (Mead and Bower, 2000), while continuity-of-care scholarship highlighted the preservation of relationships, information, and management across time (Haggerty et al., 2003). Narrative Medicine further emphasized the interpretive role of patient stories and clinical listening (Charon, 2007), integrated care frameworks addressed coordination across organizational and professional boundaries (Valentijn et al., 2013), and learning health systems emphasized the continuous movement from data to knowledge to improved care (National Academies of Sciences, Engineering, and Medicine, 2021). Despite these advances, contemporary health systems continue to experience fragmentation in how clinically relevant knowledge is represented, integrated, and carried forward across time.

Clinical Coherence is proposed as a systems-level construct that addresses this challenge. It refers to the capacity of a health system to maintain a consistent, actionable, and longitudinally meaningful representation of a patient’s condition through the integration of biological knowledge, Clinical Phenomenological Data, and interpretive clinical judgment. The concept focuses not on the quantity of information available within a system, but on the extent to which clinically relevant knowledge remains connected, interpretable, and usable across encounters, clinicians, organizations, and time.

Clinical Coherence differs from several related concepts. Continuity of care emphasizes preservation of relationships, information, and management across encounters (Haggerty et al., 2003; Pereira Gray et al., 2018). Patient-centered care emphasizes responsiveness to patient values, preferences, and needs (Mead and Bower, 2000; Epstein and Street, 2011). Narrative Medicine highlights the interpretive importance of patient stories and clinical listening (Charon, 2007). Information management focuses on the collection, storage, transmission, and retrieval of information (Farnese et al., 2019). Clinical Coherence incorporates elements of each tradition while addressing a distinct question: whether diverse forms of clinical knowledge remain sufficiently integrated to support coherent understanding and action over time.

**FIGURE 1:**
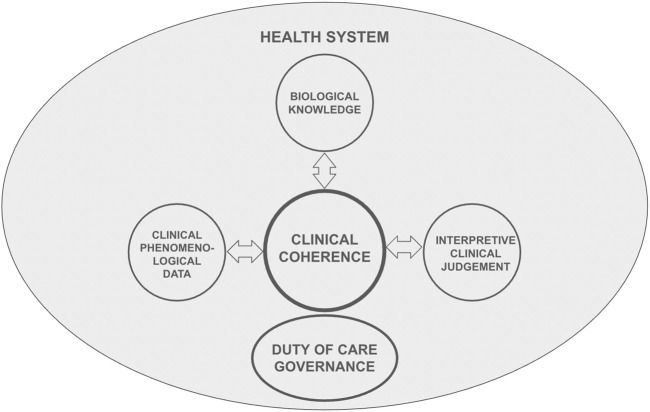
Clinical coherence as an emergent systems property. This figure illustrates Clinical Coherence as an emergent systems property arising from the integration of three interdependent domains of clinical knowledge: biological knowledge, Clinical Phenomenological Data, and interpretive clinical judgment. Clinical Phenomenological Data represents structured accounts of integrated life experiences relevant to health and wellbeing, including symptoms, function, uncertainty, family systems, spiritual or existential concerns, community context, and other experiential signals that shape how illness is perceived, interpreted, and managed. The bidirectional arrows indicate that clinically meaningful understanding emerges through integration rather than through any single domain in isolation. Duty of Care Governance supports this integration by preserving accountability, continuity, feedback, and organizational learning across the health system. Clinical Coherence is therefore represented not as an attribute of an individual clinician, encounter, or technology, but as an emergent systems property arising from the integration of these domains within a governed health system.

Within this framework, Clinical Coherence emerges through interactions among three interdependent domains of clinical knowledge: biological knowledge, Clinical Phenomenological Data, and interpretive clinical judgment (Figure 1). These domains are analytically distinct yet functionally interdependent. The preservation of meaningful relationships among them forms the basis of coherent clinical understanding. The next section specifies the domains of clinical knowledge whose integration gives rise to Clinical Coherence.

### Domains of clinical knowledge

The concept of Clinical Coherence is grounded in a longstanding recognition that clinical understanding cannot be reduced to biological information alone. Engel’s biopsychosocial model argued that illness emerges through interactions among biological, psychological, and social dimensions and therefore requires integration across multiple forms of knowledge to support effective care (Engel, 1977). Patient-centered medicine subsequently emphasized understanding illness within the patient’s lived experience and relational context (Mead and Bower, 2000; Epstein and Street, 2011), while continuity-of-care scholarship highlighted the importance of preserving relationships, information, and management across time (Haggerty et al., 2003). Narrative Medicine further emphasized the interpretive value of patient stories and clinical listening (Charon, 2007). Family systems approaches demonstrated that illness is frequently experienced and managed within relational networks rather than by individuals in isolation, while spiritual care scholarship highlighted the importance of meaning, purpose, and existential concerns in health and illness (Campbell, 2003; Puchalski et al., 2009; Koenig, 2012; Koenig, 2015). Integrated care frameworks subsequently reinforced the importance of coordination across professional, organizational, and clinical boundaries (Valentijn et al., 2013). Within the present framework, Clinical Coherence arises through the integration of three domains of clinical knowledge: biological knowledge, Clinical Phenomenological Data, and interpretive clinical judgment.

#### Biological knowledge

The biological domain encompasses laboratory findings, imaging studies, physiological measurements, genetic information, diagnostic classifications, and other forms of structured biomedical evidence that constitute the foundation of contemporary evidence-based medicine (Sackett et al., 1996; Institute of Medicine, 2001). These forms of information are typically standardized, reproducible, and readily transferable across healthcare systems. Advances in electronic health records, clinical decision-support systems, digital health technologies, and artificial intelligence have further increased the capacity of health systems to collect, store, and analyze biomedical information at scale (Adler-Milstein et al., 2017; Jiang et al., 2017; Topol, 2019; World Health Organization, 2024).

#### Clinical phenomenological data

The Clinical Phenomenological Data domain encompasses integrated life experiences relevant to health and wellbeing. These include symptoms, functional limitations, uncertainty, perceived change over time, treatment burden, illness meaning, family systems, caregiving responsibilities, spiritual or existential concerns, community influences, cultural context, work-related factors, and other environmental conditions that shape how health and illness are perceived, interpreted, and managed. This domain draws upon patient-centered medicine and Narrative Medicine, which recognize that clinically relevant knowledge cannot be fully inferred from biological measurements alone (Mead and Bower, 2000; Charon, 2007). It is also informed by patient-reported outcomes research, phenomenology, family systems theory, and spiritual care scholarship, which further emphasize symptom burden, function, meaning, relational context, and existential concerns as clinically relevant dimensions of health and illness (Cella et al., 2007; Puchalski et al., 2009; Koenig, 2012; Kotronoulas et al., 2014; Koenig, 2015; Neubauer et al., 2019). Within the present framework, these forms of knowledge are represented as Clinical Phenomenological Data when they become clinically relevant and actionable within healthcare systems. Because this domain is particularly vulnerable to fragmentation across settings and technologies, it warrants separate consideration.

#### Interpretive clinical judgment

The interpretive domain encompasses the processes through which clinicians transform biological knowledge and Clinical Phenomenological Data into coherent understanding and action. Clinical reasoning, diagnostic formulation, judgment under conditions of uncertainty, tacit professional knowledge, pattern recognition, and organizational sensemaking all contribute to this domain (Henry, 2006; Croskerry, 2009; Whitelock-Wainwright et al., 2022). Unlike biological information, interpretive knowledge is often embedded within clinicians, teams, and healthcare organizations. It represents the mechanisms through which evidence, experience, context, and uncertainty are integrated into clinically meaningful decisions.

These domains are analytically distinct but functionally interdependent. Biological findings acquire clinical significance through interpretation. Clinical Phenomenological Data influences diagnostic reasoning, therapeutic choices, prioritization of goals, and longitudinal care planning. Interpretive clinical judgment depends upon integration of biological knowledge and Clinical Phenomenological Data within a specific clinical context. Clinical Coherence emerges when these domains remain sufficiently integrated to support a consistent and clinically meaningful representation of a patient’s condition over time. Fragmentation occurs when the relationships among them become weakened across encounters, specialties, organizations, technologies, or administrative boundaries (Haggerty et al., 2003; Greenhalgh et al., 2017; Pereira Gray et al., 2018).

The vulnerability of these domains is not equal. Clinical Phenomenological Data is particularly susceptible to fragmentation and loss as healthcare becomes increasingly specialized, distributed, and technologically mediated. The next section therefore examines this challenge in greater detail.

### Clinical phenomenological data and the risk of knowledge loss

Among the domains contributing to Clinical Coherence, Clinical Phenomenological Data is particularly vulnerable to fragmentation, omission, or distortion as care becomes distributed across encounters, specialties, organizations, and technologies. In contrast to biological information, which is often standardized, reproducible, and readily transferable, many forms of experiential and contextual knowledge are embedded within relationships, conversations, observations, and longitudinal patterns of care. Patient-centered medicine and Narrative Medicine have long emphasized the clinical importance of these experiential and relational dimensions (Mead and Bower, 2000; Charon, 2007). Spiritual care scholarship further highlights how meaning, existential concerns, and relational context may shape health, coping, and engagement with care (Puchalski et al., 2009; Koenig, 2012; Koenig, 2015). As a result, these forms of knowledge are frequently more difficult to capture, transmit, and preserve across healthcare systems.

The challenge extends beyond documentation alone. Clinically relevant knowledge may be distributed across patient narratives, family systems, caregiving responsibilities, community contexts, cultural influences, spiritual or existential concerns, and evolving patterns of adaptation to illness. These dimensions often shape decision-making, engagement with treatment, coping, resilience, quality of life, and engagement with care, yet may not be fully represented within conventional medical records. Information may remain available while the context necessary for interpretation is progressively lost.

This vulnerability becomes particularly important in complex longitudinal conditions such as Primary Ovarian Insufficiency, where biological findings alone often fail to capture the full clinical reality of the condition. Reproductive concerns, uncertainty regarding future family formation, relationship dynamics, treatment burden, altered life expectations, identity disruption, social support, and spiritual or existential questions may substantially influence both patient wellbeing and clinical outcomes (Groff et al., 2005; Davis et al., 2010; Driscoll et al., 2016; Xi et al., 2023). When these dimensions are not carried forward, clinicians may inherit biological data while losing access to contextual knowledge necessary for coherent care.

Digital health technologies and artificial intelligence may both mitigate and amplify this challenge. Structured systems can improve the capture and transmission of selected forms of experiential information, yet they may preferentially preserve information that is easily measured while overlooking knowledge that is relational, contextual, narrative, or longitudinal in nature (Greenhalgh et al., 2017; Topol, 2019; World Health Organization, 2024). As health systems become increasingly dependent upon digital infrastructures, preservation of Clinical Phenomenological Data becomes a governance challenge as much as a documentation challenge.

Within the present framework, loss of Clinical Phenomenological Data represents a specific pathway through which Clinical Coherence may deteriorate. Biological information may remain intact, and clinical expertise may remain available, yet meaningful integration can still be lost when experiential and contextual knowledge become disconnected from interpretation and decision-making. This challenge illustrates why Clinical Coherence should be understood not as a property of any individual encounter, clinician, or data source, but as a systems-level condition emerging from the preservation of relationships among domains of clinical knowledge. Understanding how those relationships are maintained requires examining health systems as complex adaptive systems.

### Clinical coherence as an emergent systems property

Health systems are complex adaptive systems in which performance emerges from interactions among people, technologies, organizational structures, information flows, and clinical relationships rather than from isolated components alone (Plsek and Greenhalgh, 2001). Within such systems, Clinical Coherence is proposed as an emergent property that reflects the preservation of meaningful relationships among biological knowledge, Clinical Phenomenological Data, and interpretive clinical judgment, thereby supporting a consistent and clinically meaningful representation of a patient’s condition over time.

The concept of emergence is well established within systems science and complexity theory. Emergent properties arise through interactions among components and cannot be fully explained by examining individual elements in isolation (Plsek and Greenhalgh, 2001). Health systems similarly exhibit characteristics that are not reducible to individual clinicians, technologies, or organizational units. Continuity of care, organizational learning, safety culture, and system resilience similarly depend upon interactions occurring across multiple levels of a system (Scally and Donaldson, 1998; Chassin and Loeb, 2013; National Academies of Sciences, Engineering, and Medicine, 2021).

Clinical Coherence operates in a similar manner. A health system may possess extensive biological data, sophisticated digital infrastructure, highly trained clinicians, and substantial organizational resources while still failing to maintain a coherent understanding of a patient’s condition over time. Conversely, coherent care may emerge when diverse forms of clinical knowledge remain effectively integrated despite organizational complexity. The critical determinant is therefore not the volume of information available, but the degree to which knowledge remains connected, interpretable, and actionable across time and context.

This Perspective distinguishes Clinical Coherence from related concepts such as continuity of care, patient-centered care, and information management. Continuity emphasizes preserving relationships and information across encounters (Haggerty et al., 2003; Pereira Gray et al., 2018). Patient-centered care emphasizes responsiveness to patient values, needs, and preferences (Mead and Bower, 2000; Epstein and Street, 2011). Information management focuses on the collection, storage, retrieval, and transmission of data (Farnese et al., 2019). Clinical Coherence incorporates elements of each but focuses specifically on whether clinically meaningful knowledge remains integrated across domains and over time. Information may be continuous yet poorly integrated. Care may be patient-centered yet fragmented across specialties. Data may be available yet insufficiently connected to clinical understanding.

From a systems perspective, coherence and incoherence exist along a continuum rather than as binary states. Health systems may preserve strong integration in some domains while exhibiting fragmentation in others. Similarly, coherence may vary across conditions, organizations, care pathways, and patient populations. This formulation therefore does not assume that complete coherence is achievable in all circumstances. Instead, it proposes that the degree of coherence influences a system’s capacity to translate information into clinically meaningful understanding and action.

Viewed in this way, Clinical Coherence represents neither a new data source nor a new category of clinical intervention. Rather, it describes a systems-level condition that influences whether existing forms of clinical knowledge can be translated into coherent longitudinal care. The framework therefore shifts attention from the quantity of information available within health systems to the quality of integration among the domains of knowledge that support clinical understanding.

Donabedian’s observation that healthcare quality emerges through relationships among structures, processes, and outcomes provides a useful parallel (Donabedian, 1988). Clinical Coherence extends this systems perspective by focusing specifically on preservation of meaningful relationships among domains of clinical knowledge. In this formulation, coherence is not synonymous with quality, continuity, or patient-centeredness, although it may contribute to each. Rather, it reflects the degree to which clinically relevant knowledge remains sufficiently integrated to support a stable and actionable understanding of the patient across time, settings, and organizational boundaries.

If Clinical Coherence is an emergent systems property, it must ultimately be studied through observable patterns of integration, fragmentation, continuity, and knowledge preservation across the patient journey. This framing therefore creates the basis for operationalizing Clinical Coherence.

#### Operationalizing clinical coherence

For Clinical Coherence to function as a useful systems-level construct, it must be capable of operational definition, empirical investigation, and eventual measurement. The purpose of this paper is not to propose a fully validated instrument, but to establish a conceptual foundation from which measurable indicators can be developed and tested. Consistent with established approaches to health systems measurement, Clinical Coherence is conceptualized as an emergent property that can be inferred from observable patterns of knowledge integration rather than measured directly through a single variable (Donabedian, 1988; Mokkink et al., 2010).

Within this framework, the primary unit of analysis is the longitudinal patient journey. While coherence may be examined at the level of individual encounters, clinicians, teams, organizations, or health systems, its consequences become most visible across time as patients move through multiple episodes of care. Clinical Coherence therefore reflects the extent to which clinically meaningful knowledge remains integrated, interpretable, and actionable throughout the course of care.

Potential indicators of Clinical Coherence may be examined within and across domains of clinical knowledge. Examples include preservation of clinically relevant information across encounters, continuity of contextual understanding, consistency of diagnostic representation over time, alignment between patient-reported experience and clinical interpretation, continuity of management strategies, and preservation of meaningful context during transitions of care. At a systems level, coherence may also be reflected in the degree to which biological knowledge, Clinical Phenomenological Data, and interpretive clinical judgment remain connected across organizational boundaries.

Conversely, incoherence may be reflected through recurrent patterns of fragmentation. Potential indicators include repeated reconstruction of patient history, contradictory interpretations across encounters, loss of clinically relevant context, unexplained diagnostic delay, inconsistent management approaches, and progressive divergence between patient-reported experience and documented clinical understanding. These patterns do not necessarily indicate deficiencies within individual clinicians or organizations; rather, they may reflect failures in the preservation and integration of clinical knowledge across distributed systems of care.

Importantly, Clinical Coherence is not proposed as a replacement for existing measures of continuity, patient-reported outcomes, quality, or health system performance. Rather, it provides a framework for examining how these measures relate to one another through the preservation, or loss, of clinically meaningful integration across domains of knowledge.

Future work should focus on developing and validating measurement approaches capable of assessing Clinical Coherence across patient journeys, organizations, and health systems. Such efforts may include longitudinal pathway analysis, continuity measures, patient-reported assessments, mixed-methods evaluation, network analysis, implementation science methodologies, and health system performance indicators. Particular attention should be directed toward establishing construct validity, discriminant validity relative to related concepts such as continuity of care and integrated care, and responsiveness to change across clinical settings (Mokkink et al., 2010).

Establishing reliable approaches to measurement represents a necessary next step for evaluating the framework’s relevance to healthcare governance, digital health, artificial intelligence, quality improvement, and learning health systems. Sustaining these patterns of integration ultimately depends upon governance structures capable of maintaining responsibility, escalation, feedback, and continuity across organizational boundaries.

### Duty of care governance as the structural architecture for clinical coherence

**FIGURE 2:**
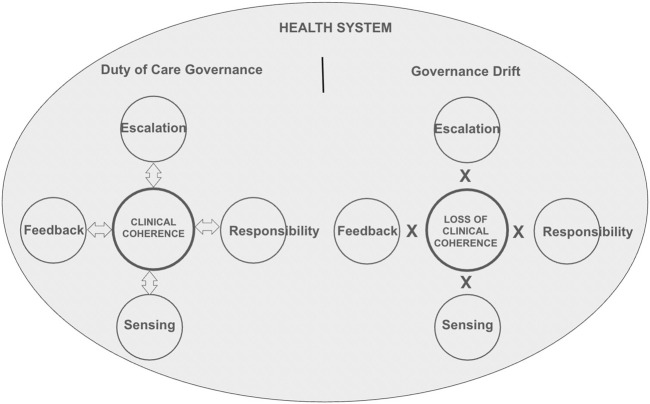
Duty of care governance as the structural architecture of clinical coherence. This figure illustrates Duty of Care Governance as the organizational architecture through which Clinical Coherence is preserved within health systems. On the left, Duty of Care Governance is depicted as an integrated governance configuration in which Sensing, Responsibility, Escalation, and Feedback remain connected to Clinical Coherence through bidirectional relationships. These connections indicate that clinical signals can be recognized, interpreted, acted upon, escalated when necessary, and reconnected to outcomes through feedback and learning. On the right, Governance Drift is depicted as a contrasting configuration in which these relationships become attenuated or disrupted. The X marks indicate weakened or broken connections among Sensing, Responsibility, Escalation, Feedback, and the central representation of the patient’s condition. Under these conditions, clinically relevant knowledge may become fragmented across organizational, technological, or administrative layers, resulting in loss of Clinical Coherence despite the continued availability of information. Together, the two configurations illustrate that Clinical Coherence depends not only on the presence of information, but on governance structures capable of preserving meaningful relationships among clinical signals, responsibility, escalation, feedback, interpretation, and organizational learning over time. Loss of Clinical Coherence is therefore represented not primarily as a failure of information availability, but as a failure to preserve the relationships through which clinical knowledge remains meaningful and actionable.

Within the present framework, Duty of Care Governance is proposed as the structural architecture through which Clinical Coherence is preserved (Figure 2). The framework builds upon established traditions in healthcare quality, systems theory, continuity of care, knowledge management, clinical governance, and complex adaptive systems. Rather than introducing an entirely new theory of healthcare, it extends a longstanding scientific effort to understand how clinically meaningful knowledge can be preserved as health systems increase in scale, specialization, and complexity.

Foundational work by Donabedian established that healthcare quality emerges through relationships among structures, processes, and outcomes rather than through isolated components alone (Donabedian, 1988). Complexity science subsequently demonstrated that health systems function as adaptive networks whose behavior emerges through interactions occurring across organizational and temporal scales (Plsek and Greenhalgh, 2001). Continuity-of-care scholarship further highlighted the importance of preserving relationships, information, and management over time as essential determinants of effective healthcare delivery (Haggerty et al., 2003; Pereira Gray et al., 2018). Together, these traditions established a common principle: health system performance depends not only on the quality of individual components, but also on the integrity of the relationships among them.

Building upon this foundation, clinical governance emphasized accountability and quality improvement (Scally and Donaldson, 1998; Chassin and Loeb, 2013), while knowledge-management scholarship examined how information becomes actionable understanding within organizations (Farnese et al., 2019). Recent work on Duty of Care Governance, safe harboring, and multiscale coherence has explored how accountability, vigilance, escalation pathways, relational continuity, and organizational learning can remain aligned as health systems scale (Nelson, 2026a; Nelson, 2026b; Nelson, 2026c).

Clinical Coherence and Duty of Care Governance address related but distinct questions. Clinical Coherence describes the systems-level condition in which clinically meaningful knowledge remains sufficiently integrated to support coherent understanding and action over time. Duty of Care Governance describes the organizational architecture through which that integration is preserved. In this formulation, Clinical Coherence represents the condition to be preserved, whereas Duty of Care Governance represents the mechanism through which health systems preserve it.

Clinical Coherence cannot be sustained through information systems alone. Preservation of clinically meaningful knowledge requires governance structures capable of maintaining accountability, continuity, responsibility, escalation, and feedback across organizational boundaries. This challenge has become increasingly important as health systems have grown in scale, specialization, and technological complexity, creating conditions in which clinically relevant knowledge may become separated from decision-making despite the continued availability of information.

Governance has traditionally been understood as the set of structures, processes, and relationships through which organizations establish accountability, allocate authority, manage risk, and pursue quality improvement (Scally and Donaldson, 1998; Chassin and Loeb, 2013). Within healthcare, governance systems influence which information is recognized, how concerns are escalated, who holds responsibility for action, and how feedback is incorporated into organizational learning. As health systems scale, these governance functions increasingly determine whether clinically meaningful knowledge remains connected to decision-making or becomes fragmented across administrative, technological, and organizational layers.

The framework is organized around four interdependent functions: Sensing, Responsibility, Escalation, and Feedback. Sensing refers to the capacity of health systems to recognize clinically relevant signals arising from biological findings, Clinical Phenomenological Data, and frontline clinical observation. Responsibility refers to the assignment of clear accountability for interpreting and responding to those signals. Escalation provides pathways through which concerns that exceed local capacity can move to higher levels of organizational awareness and action. Feedback ensures that decisions and interventions remain connected to outcomes, allowing organizations to learn, adapt, and refine their understanding over time.

These functions operate together to reduce the risk of Governance Drift, a condition in which organizational decision-making becomes progressively separated from clinical reality. Under conditions of Governance Drift, patient experience may be weakly represented, clinically relevant concerns may fail to reach decision-makers, and institutional priorities may become increasingly disconnected from frontline care. The result is often fragmentation not because information is absent, but because relationships among knowledge, responsibility, and action have deteriorated.

The importance of governance becomes particularly evident within digitally enabled healthcare environments. Electronic health records, artificial intelligence systems, predictive analytics, and large-scale data infrastructures can substantially increase the availability of information. However, information alone does not guarantee coherent care. Without governance structures capable of maintaining meaningful connections among biological knowledge, Clinical Phenomenological Data, and interpretive clinical judgment, health systems may accumulate information while simultaneously losing coherence (Floridi et al., 2018; Amann et al., 2020; Lekadir et al., 2025). The challenge is therefore not merely technological but organizational and epistemic.

From this perspective, Clinical Coherence and Duty of Care Governance operate as complementary constructs. Clinical Coherence describes whether clinically meaningful knowledge remains integrated. Duty of Care Governance describes the organizational mechanisms through which that integration is preserved, monitored, and restored when threatened. Together, they provide a framework for understanding how health systems can maintain coherent clinical knowledge as care becomes increasingly distributed across people, organizations, and technologies.

Accordingly, preservation of Clinical Coherence should be understood not only as a clinical objective but also as a governance responsibility. Health systems that successfully maintain alignment among clinical signals, interpretive judgment, organizational decision-making, and patient experience are more likely to sustain coherent longitudinal care despite increasing complexity and scale. In this formulation, governance serves not merely to regulate healthcare delivery, but to preserve the relationships that allow clinical knowledge to remain meaningful and actionable over time.

### Primary ovarian insufficiency as a sentinel condition

Sentinel conditions have long been used in health services research and quality improvement as indicators of broader system performance because they make underlying weaknesses in care delivery more visible (Donabedian, 1988; Institute of Medicine, 2001). Within the present framework, Primary Ovarian Insufficiency serves as a sentinel condition for examining Clinical Coherence because effective care depends upon the sustained integration of biological knowledge, Clinical Phenomenological Data, and interpretive clinical judgment across extended periods of uncertainty, longitudinal follow-up, and multidisciplinary care.

Primary Ovarian Insufficiency is characterized by impaired ovarian function before the age of forty and is associated with infertility, menstrual irregularity, hypoestrogenism, and increased long-term health risks affecting bone, cardiovascular, and psychosocial wellbeing (Nelson, 2009; Panay et al., 2024). Although the biological foundations of ovarian function and reproductive endocrinology are increasingly well understood, women frequently experience substantial delays in diagnosis, fragmented care pathways, inconsistent clinical messaging, and inadequate continuity across healthcare encounters (Alzubaidi et al., 2002; Groff et al., 2005; Davis et al., 2010; Xi et al., 2023).

The condition illustrates the interdependence of multiple domains of clinical knowledge. Biological knowledge includes hormonal measurements, reproductive history, genetic findings, autoimmune evaluation, and long-term health monitoring. Clinical Phenomenological Data includes menstrual changes, fertility concerns, uncertainty, treatment burden, altered life expectations, family formation concerns, psychosocial adaptation, and other contextual factors influencing health and wellbeing. Interpretive clinical judgment is required to integrate these signals into coherent understanding, diagnosis, and management. No single domain alone is sufficient to represent the condition adequately.

Failures of integration become readily visible in this context. Women may undergo repeated evaluations without recognition of emerging diagnostic patterns, receive conflicting explanations from different clinicians, or repeatedly reconstruct their history as care transitions across specialties and organizations (Groff et al., 2005; Driscoll et al., 2016; Xi et al., 2023). Biological findings may be available while contextual knowledge remains insufficiently incorporated into clinical reasoning. Conversely, experiential concerns may be acknowledged without adequate integration into longitudinal care planning. The result is often not a lack of information, but fragmentation in how information is connected, interpreted, and acted upon over time.

The recent international guideline emphasizes the importance of coordinated, multidisciplinary, and longitudinal approaches to care for women with Primary Ovarian Insufficiency, reflecting recognition that successful management extends beyond isolated diagnostic or therapeutic interventions (Panay et al., 2024). The condition therefore provides a useful illustration of the broader challenge addressed by Clinical Coherence: whether health systems can preserve meaningful integration among biological knowledge, Clinical Phenomenological Data, and interpretive clinical judgment as care becomes increasingly specialized and distributed.

Primary Ovarian Insufficiency is not unique in this regard. Similar challenges arise across many chronic, multisystem, and diagnostically complex conditions. However, it offers a particularly informative example because fragmentation often becomes visible simultaneously across reproductive, endocrine, psychosocial, and long-term preventive domains. As such, it serves as a sentinel condition through which the presence, or absence, of Clinical Coherence may be more readily observed.

Viewed through this lens, Primary Ovarian Insufficiency demonstrates that the central challenge for modern health systems is not simply generating more information, but preserving the relationships among domains of clinical knowledge that allow information to become clinically meaningful over time. These challenges are likely to become increasingly consequential as digital health and artificial intelligence reshape how clinical knowledge is represented, interpreted, and acted upon.

## Implications for digital health and artificial intelligence

The rapid expansion of digital health technologies and artificial intelligence has intensified longstanding questions regarding how clinical knowledge is represented, integrated, and applied within healthcare systems. Advances in electronic health records, predictive analytics, machine learning, remote monitoring, and clinical decision-support systems have substantially increased the capacity of health systems to collect, process, and analyze information at scale (Adler-Milstein et al., 2017; Topol, 2019). Recent guidance on artificial intelligence for health further emphasizes the need for responsible, trustworthy, and deployable systems as these technologies become embedded within healthcare (World Health Organization, 2024; Lekadir et al., 2025). These technologies hold considerable promise for improving diagnostic accuracy, identifying patterns across large datasets, supporting clinical decision-making, and expanding access to healthcare knowledge. At the same time, they introduce new challenges regarding preservation of clinically meaningful integration across domains of knowledge.

Artificial intelligence systems operate on representations of reality rather than reality itself. Their performance depends upon the quality, completeness, structure, and contextual integrity of the information available to them (Patel et al., 2009; Floridi et al., 2018; Amann et al., 2020). As a result, the effectiveness of artificial intelligence is influenced not only by algorithmic sophistication, but also by the coherence of the clinical knowledge environment from which it learns and upon which it acts.

From the perspective of Clinical Coherence, the central question is not whether artificial intelligence can process increasing quantities of information, but whether clinically meaningful relationships among domains of knowledge remain preserved. Systems that receive fragmented representations of patients may generate fragmented outputs regardless of computational power. Conversely, systems operating within coherent knowledge environments may be better positioned to support clinically meaningful interpretation, continuity, and decision-making.

Clinical Coherence therefore provides a potential framework for evaluating trustworthy artificial intelligence in healthcare. Recent international initiatives have emphasized transparency, explainability, accountability, human oversight, and responsible deployment of artificial intelligence systems (Floridi et al., 2018; World Health Organization, 2021; Lekadir et al., 2025). Clinical Coherence complements these efforts by focusing attention on the integrity of the underlying knowledge environment. Such systems require not only reliable algorithms, but also preservation of meaningful relationships among biological knowledge, Clinical Phenomenological Data, and interpretive clinical judgment.

This perspective has practical implications for health system design. Electronic health records, patient portals, remote monitoring platforms, and artificial intelligence applications should be evaluated not only according to efficiency, predictive performance, or data-processing capacity, but also according to their ability to preserve integration across domains of clinical knowledge. Systems that successfully process biological information while eroding contextual understanding or interpretive continuity may increase informational volume without improving coherent care. Conversely, technologies designed to strengthen integration across domains may enhance both clinical understanding and organizational learning.

Clinical Coherence may also provide a conceptual bridge between learning health systems and emerging artificial intelligence governance frameworks. Learning health systems seek to transform data into knowledge and knowledge into improved care (Institute of Medicine, 2001; National Academies of Sciences, Engineering, and Medicine, 2021). Clinical Coherence extends this objective by emphasizing that the value of learning depends upon preservation of the relationships among the domains of knowledge being learned from. Data alone does not create understanding. Understanding emerges through integration of biological knowledge, Clinical Phenomenological Data, and interpretive clinical judgment within systems capable of maintaining those relationships over time.

As healthcare becomes increasingly dependent upon digital infrastructures and artificial intelligence, preserving Clinical Coherence may become as important as improving computational capability itself. The future challenge is not merely to build systems that know more, but to build systems that preserve the conditions through which knowledge becomes understanding and understanding becomes care.

## Strengths, limitations, and future directions

This framework has several strengths. First, it synthesizes insights from multiple established traditions, including the biopsychosocial model, patient-centered medicine, Narrative Medicine, continuity of care, integrated care, systems theory, complexity science, knowledge management, and clinical governance, into a unified framework focused on preservation of clinically meaningful knowledge across scalable health systems (Engel, 1977; Donabedian, 1988; Plsek and Greenhalgh, 2001; Haggerty et al., 2003; Charon, 2007; Valentijn et al., 2013; Farnese et al., 2019). Rather than introducing an entirely new theory of healthcare, Clinical Coherence integrates existing traditions around a common challenge: preserving meaningful relationships among domains of clinical knowledge over time.

Second, the framework addresses a growing challenge within contemporary healthcare. As health systems become increasingly digital, distributed, and data intensive, the capacity to generate information often exceeds the capacity to preserve meaningful integration among forms of clinical knowledge. By focusing on knowledge integration rather than information accumulation alone, Clinical Coherence provides a conceptual bridge linking clinical practice, governance, implementation science, digital health, and artificial intelligence.

Third, the framework establishes a foundation for empirical investigation. By identifying clinically meaningful integration as a systems-level phenomenon, it provides a structure through which patterns of coherence and fragmentation may be examined across patient journeys, organizations, and health systems.

Several limitations should also be acknowledged. Most importantly, Clinical Coherence remains a conceptual framework. Although this manuscript proposes candidate domains, mechanisms, and indicators, the construct has not yet undergone formal validation and should therefore be regarded as hypothesis generating. Future work will be required to establish reliability, validity, discriminant validity, and practical utility across diverse healthcare settings.

A second limitation concerns overlap with related constructs. While Clinical Coherence is proposed as addressing a distinct knowledge-integration problem, empirical investigation will be necessary to determine its explanatory value beyond existing frameworks such as continuity of care, integrated care, patient-centered care, and learning health systems.

A third limitation is that the framework is illustrated primarily through Primary Ovarian Insufficiency. Although the condition provides a useful sentinel example, future studies should examine whether Clinical Coherence is similarly relevant across other chronic, multisystem, and diagnostically complex conditions.

Several priorities for future research emerge from this work. The first involves development and validation of measurement approaches capable of assessing Clinical Coherence across patient journeys, organizations, and health systems. Particular attention should be directed toward establishing construct validity, discriminant validity relative to related concepts, and responsiveness to change over time (Mokkink et al., 2010).

A second priority involves testing the framework across diverse clinical conditions and care environments. Such work would help determine the extent to which Clinical Coherence represents a generalizable systems construct rather than one primarily applicable to specific disease categories.

A third priority concerns digitally enabled healthcare environments. Future studies should examine whether electronic health records, patient portals, clinical decision-support systems, and artificial intelligence applications strengthen or weaken Clinical Coherence and identify design principles that promote preservation of clinically meaningful integration across domains of knowledge.

A fourth area for investigation concerns governance. Future research should evaluate whether governance structures emphasizing sensing, responsibility, escalation, and feedback are associated with improved preservation of Clinical Coherence across organizational boundaries. Such work could help clarify relationships among governance design, knowledge integration, organizational learning, and health system performance.

Finally, future scholarship should examine associations between Clinical Coherence and outcomes relevant to patients, clinicians, organizations, and health systems, including trust, continuity, resilience, quality of care, and organizational learning. If the framework proves useful empirically, Clinical Coherence may provide a novel lens through which health systems can evaluate their capacity not merely to collect information, but to preserve the conditions under which information becomes clinically meaningful.

Taken together, these strengths, limitations, and future directions suggest that Clinical Coherence should be viewed as an emerging systems-level construct whose ultimate value will depend upon empirical testing, practical application, and continued refinement across diverse healthcare contexts.

## Conclusion

Clinical Coherence reframes health system performance as the capacity to preserve meaningful integration among biological knowledge, Clinical Phenomenological Data, and interpretive clinical judgment. As care becomes increasingly specialized, distributed, and digitally mediated, the central challenge is not simply generating more information, but maintaining the relationships that allow information to become clinically meaningful.

Duty of Care Governance provides a structural architecture for preserving this integration by linking clinical signals to responsibility, escalation, feedback, organizational learning, and decision-making. Primary Ovarian Insufficiency illustrates how fragmentation across domains of clinical knowledge can undermine care even when substantial biomedical knowledge is available.

As digital health and artificial intelligence continue to reshape healthcare, Clinical Coherence offers a framework for evaluating whether health systems preserve the conditions through which information becomes knowledge, knowledge becomes understanding, and understanding becomes care. In this formulation, the future challenge is not merely building systems that know more, but building systems that preserve the integrity of the relationships that make knowledge clinically meaningful. Clinical Coherence therefore provides a lens through which healthcare organizations may evaluate their capacity to sustain meaningful understanding across patients, clinicians, technologies, and time.

## Data Availability

The original contributions presented in the study are included in the article/supplementary material, further inquiries can be directed to the corresponding author.
